# Short-video content perceptions, intention, and self-reported behavior in special forces-style tourism: Evidence from Chinese generation Z university students

**DOI:** 10.1371/journal.pone.0361990

**Published:** 2026-11-03

**Authors:** Hongjing Mo, Min Wei, Jingyu Liu

**Affiliations:** Faculty of Economics and Management, Changsha Normal University, Changsha, Hunan Province, China; Guangxi Normal University, CHINA

## Abstract

Special Forces-style tourism is an emerging high-intensity, low-cost travel phenomenon among young people in China. Drawing on a modified Theory of Planned Behavior, this study analyzes survey data from 526 Chinese Generation Z university students using PLS-SEM and fsQCA. Perceived content appeal, perceived content trustworthiness, attitude, and perceived behavioral control are positively associated with intention, which is positively associated with self-reported behavior; short-video platform usage intensity strengthens this association. fsQCA identifies two configurations for high and eight for low self-reported behavior. Intention, perceived content trustworthiness, perceived behavioral control, and platform usage intensity form the common core of both high-outcome configurations, while low perceived content trustworthiness appears in six low-outcome configurations. No single condition is necessary, and the results indicate configurational asymmetry.

## 1. Introduction

Special Forces-style tourism (SFT) is a high-intensity form of youth travel in China characterized by short durations, compressed itineraries, limited expenditure, and intensive visits to multiple attractions or destinations [1,2]. It emphasizes efficiency, physical effort, and self-challenge, and has been associated with identity expression and social recognition among younger travelers [3,4]. The China Tourism Academy identifies it as an emerging form of domestic tourism and uses the term ‘special forces tourism’ in its English-language material [5]. Following recent peer-reviewed studies [2,6], this study uses ‘Special Forces-style tourism’, abbreviated as SFT.

The emergence of SFT is closely connected to digital media. Short-video and social-media platforms circulate user-generated information and visual travel narratives that inform tourism decisions [7–9]. For SFT, such content can communicate both the appeal and the practical demands of compressed, low-cost itineraries [2,6]. Potential participants may therefore evaluate not only how engaging the content is, but also whether its descriptions of routes, costs, schedules, and physical demands provide a credible basis for planning. These content evaluations offer a useful starting point for examining SFT intention in a short-video environment.

Chinese Generation Z is particularly relevant to this setting because digital media are prominent in young travelers’ information search, self-presentation, and experience sharing [7–9]. Research on urban Chinese travelers aged 18–35 identifies both SFT and ‘lying-flat’ leisure travel as characteristic youth travel orientations, illustrating the diversity of preferences within this broader age group [10]. SFT studies further connect intensive travel with experience accumulation, self-expression, and visible accomplishment among young people [4,9,11]. Qualitative evidence shows how social media inspires and circulates SFT experiences while providing opportunities for identity expression [6].

Within Generation Z, this study focuses on university students. A preliminary estimate suggests that students account for more than 85% of SFT participants [12], making them an important group for examining the phenomenon. Their budget constraints and interest in intensive, low-cost experiences are also relevant to SFT [9,11]. The findings are therefore interpreted within the university-student population; broader sampling limitations are considered in Section 5.5.

Existing SFT research has examined its characteristics, antecedents, peer diffusion, media visibility, embodied practices, symbolic consumption, social comparison, identity, and motivations [1,4,9,11,13]. Much of this work is qualitative; for example, Zhang and Yang [6] explored authenticity through participants’ experiences of practical constraints and digital space. Quantitative research includes Zhao et al.’s [2] examination of supportive social-media communication and SFT intention. However, the joint roles of short-video content perceptions, evaluations of participating in SFT, and perceived behavioral feasibility remain insufficiently understood. Perceived content appeal (PCA) concerns how engaging and useful the content is, whereas perceived content trustworthiness (PCT) concerns its authenticity and reliability [14–17]. These perceptions are distinct from attitude toward participating in SFT and perceived behavioral control (PBC), creating a need to examine their associations with intention within a common framework.

A second issue concerns the relationship between intention and self-reported behavior (BEH). The Theory of Planned Behavior (TPB) distinguishes motivational readiness from behavioral engagement and recognizes that opportunities and constraints can affect their correspondence [18,19]. In this study, intention captures future plans and willingness, whereas BEH captures previous engagement in, and tendencies toward, SFT-related travel patterns. Examining their association can extend research beyond participation intention, although the cross-sectional design does not establish that current intention preceded the reported behavior. Short-video platform usage intensity (SVPUI) is examined as a contextual factor that may vary with the strength of this association.

TPB [18] provides the theoretical starting point and has been widely applied in tourism and hospitality [20]. Tourism studies have also adapted its specification to particular research settings [21–23]. The present modified framework includes attitude and PBC alongside PCA and PCT, and examines SVPUI as a moderator of the intention–BEH relationship. Subjective norm was not measured. The study consequently tests relationships among selected TPB and digital-media constructs, with the implications of omitting normative social influence addressed in Sections 2.1 and 5.5.

The study also examines whether different combinations of conditions are associated with similar levels of self-reported behavior. Partial Least Squares Structural Equation Modeling (PLS-SEM) estimates average net relationships and the SVPUI interaction, while fuzzy-set Qualitative Comparative Analysis (fsQCA) identifies combinations associated with high and low outcome levels. Their combined use enables the examination of equifinality and configurational asymmetry, including whether combinations associated with low outcome levels differ from simple reversals of those associated with high outcome levels [24].

The study thus examines how short-video content evaluations, attitude, and perceived feasibility relate to SFT intention, and how intention and platform engagement relate to self-reported behavior. It contributes by distinguishing content-level evaluations from evaluations of the travel activity, examining how platform engagement relates to this association, and identifying alternative configurations associated with high and low outcome levels. Together, these perspectives provide a more specific account of SFT-related intentions and reported travel patterns among Chinese Generation Z university students.

Accordingly, the study addresses the following research questions:

RQ1: To what extent are perceived content appeal, perceived content trustworthiness, attitude, and perceived behavioral control positively associated with Chinese Generation Z university students’ intention to engage in Special Forces-style tourism?
RQ2: Is intention to engage in Special Forces-style tourism significantly associated with self-reported behavior?
RQ3: Does short-video platform usage intensity moderate the relationship between intention and self-reported behavior?
RQ4: What configurations of perceived content appeal, perceived content trustworthiness, attitude, perceived behavioral control, intention, and short-video platform usage intensity are associated with high and low levels of self-reported SFT-related behavior?

## 2. Literature review and hypothesis development

### 2.1 Theory of planned behavior and its contextual modification

The Theory of Planned Behavior (TPB) is a well-established framework for explaining the relationship between intention and behavior and has been widely applied in tourism research [18,20,25]. In the original TPB, intention is primarily shaped by three conceptually distinct determinants: Attitude, Subjective Norm, and Perceived Behavioral Control (PBC). Intention is subsequently related to behavior. Attitude refers to an individual’s overall favorable or unfavorable evaluation of performing a behavior. Subjective Norm concerns perceived approval, expectations, or normative pressure from salient social referents regarding whether the individual should perform that behavior. PBC reflects the individual’s judgment of whether the capabilities, resources, and opportunities required to perform the behavior are available [18].

In the present study, these constructs are defined specifically in relation to Special Forces-style tourism (SFT). Attitude refers to individuals’ overall evaluation of participating in SFT, including whether this intensive and highly compressed form of travel is regarded as enjoyable, valuable, and worthwhile. PBC refers to individuals’ judgments of whether they possess sufficient time, financial resources, physical capacity, and ability to manage the travel arrangements required for SFT. Intention represents individuals’ future plans, willingness, and readiness to participate in SFT. Self-reported behavior (BEH) captures respondents’ reported engagement in and tendency toward SFT-related travel patterns: a fast-paced trip in the past year (BEH1), a tendency to plan intensive itineraries (BEH2), participation in physically demanding travel activities (BEH3), and past experiences involving tight schedules and high-intensity arrangements (BEH4). It is therefore a multi-item measure of reported travel patterns rather than a count of completed SFT trips.

Tourism research has extended TPB with constructs such as travel motivation, destination image, satisfaction, and other context-specific factors [19,26–29]. Recent empirical studies have integrated TPB with the elaboration likelihood model to examine short-video content and city travel intention [30], and with electronic word-of-mouth and hotel image to examine hotel purchase intention [31]. These applications illustrate the relevance of digital information to tourism and hospitality decisions. The present study incorporates three digital-media constructs: PCA captures the appeal and informational usefulness of SFT-related short videos; PCT captures their perceived authenticity, reliability, and informational credibility; and SVPUI captures engagement with short-video platforms and tourism-related content. PCA and PCT concern evaluations of content, while attitude concerns evaluation of participating in SFT itself. SVPUI is examined as a moderator of the intention–BEH relationship.

The model focuses on behavioral evaluation and perceived feasibility alongside short-video content perceptions and platform engagement. This focus explains the selection of attitude, PBC, PCA, PCT, and SVPUI, but does not establish that subjective norm can be omitted without consequences. Friends’ and family members’ approval and expectations may be relevant to SFT decisions, consistent with the role of normative influence in TPB [18]. Because subjective norm was not measured, the present data cannot establish whether its omission is empirically justified or whether its inclusion would change the estimated relationships. PCA, PCT, and SVPUI are not substitutes for subjective norm. The modified framework therefore tests relationships among selected TPB and digital-media constructs rather than the complete TPB model. The omission is explicitly acknowledged as a limitation of model specification in Section 5.5.

### 2.2 Perceived content appeal and intention

Perceived content appeal (PCA) refers to the affective and cognitive attraction elicited by short-video content, encompassing emotional resonance, visual vividness, narrative immersion, and informational utility [16,17]. Within the modified TPB framework adopted here, PCA occupies a different analytical level from attitude. Attitude is a global evaluative judgment about performing a behavior, whereas PCA operates at the content level as an immediate perceptual response to the specific audiovisual and informational features encountered during viewing. The distinction lies in the referent of evaluation: PCA captures how compelling and useful the content itself is perceived to be, whereas Attitude captures how desirable participation in SFT is considered. Short-video research has similarly distinguished evaluations of video content from evaluations of the tourism activity or destination itself [32].

Existing research indicates that content appeal relates to intention through both affective and cognitive routes. On the affective side, emotionally engaging content attracts attention, elicits emotional responses, and strengthens users’ interest in the experiences portrayed [16,33]. Experimental evidence further shows that the visual perspective and content orientation of short videos shape travel intention by enhancing imagery fluency and affect intensity, indicating that content features operate on intention through identifiable psychological mechanisms rather than mere covariation [34]. Emotional states elicited by short videos have likewise been found to mediate the relationship between content features and audience attitudes [32]. On the cognitive side, informativeness and perceived usefulness supply the basis on which users evaluate and imagine prospective travel experiences [17,35]. Incorporating content-level evaluations into a TPB framework also has recent precedent: combining TPB with the elaboration likelihood model, short-video content features have been shown to shape city travel intention through attitude [30]. Taken together, PCA represents an overall content-level evaluation rather than aesthetic appeal alone.

These arguments may also apply to SFT. Research connects this travel form with intensive itineraries, constrained resources, and digitally mediated experiences [1,6,9,11]. Tourism short-video studies show that emotional appeal, visual presentation, and useful information can contribute to travel-related responses and intentions [16,17,34,35]. In the SFT context, engaging content may make an intensive trip appear worth attempting, while practical information may help viewers imagine how to organize it. These are proposed explanations for the expected association, rather than mechanisms separately tested in the present study. We therefore expect viewers who perceive SFT-related content as engaging and useful to report stronger participation intention. Accordingly, the following hypothesis is proposed:

H1: Perceived content appeal is positively associated with Chinese Gen Z university students’ intention to engage in Special Forces-style tourism.

### 2.3 Perceived content trustworthiness and intention

Perceived content trustworthiness (PCT) refers to viewers’ assessment of the authenticity, objectivity, and informational reliability of short-video content [14,15]. Credibility carries particular weight in digital tourism environments, where users rely extensively on consumer-generated information when evaluating destinations and travel options, and where judgments of informational reliability determine whether such content is accepted and used in decision-making [36]. PCT is conceptually distinct from Attitude because the former concerns the credibility of short-video content, whereas the latter concerns individuals’ evaluation of participating in SFT itself. PCT is therefore treated as a media-specific content perception within the modified TPB framework.

Existing research broadly supports a positive relationship between credibility-related perceptions and tourism responses. Perceived authenticity and online review information have been associated with travelers’ trust and tourism-related intentions [15,37], and the combination of authenticity, informativeness, and social endorsement within user-generated content contributes to tourism engagement [38]. Research on travel vlogs provides a more specific explanation: in travel vlog settings, the sincerity and professionalism of a content source shape travel planning behavior through inspiration as a mediator, indicating that credibility operates not merely by adding information but by altering viewers’ psychological engagement with the content [39]. In short-video settings specifically, content authenticity has been shown to shape audiences’ attitudes toward both the video and the destination it portrays [32].

Credibility may be particularly relevant to SFT because planning compressed itineraries requires judgments about time, costs, and physical demands [1,6]. Research on digital tourism information links trust with the adoption of online recommendations [36], while research on misleading short videos indicates that content manipulation can weaken user trust [40]. In SFT, inaccurate itinerary or cost information may make a proposed trip appear less feasible. These considerations suggest that content perceived as realistic and reliable may provide a stronger basis for participation intention. Accordingly, the following hypothesis is proposed:

H2: Perceived content trustworthiness is positively associated with Chinese Gen Z university students’ intention to engage in Special Forces-style tourism.

### 2.4 Perceived behavioral control and intention

Perceived behavioral control (PBC) refers to individuals’ subjective assessment of the capacity and resources required to perform a behavior, encompassing both internal efficacy beliefs and perceptions of external facilitating conditions [18]. In the present context, PBC refers specifically to individuals’ judgments of whether they possess sufficient time, financial resources, physical capacity, and control over travel arrangements to undertake this intensive form of travel. These dimensions correspond to the constraint types identified in leisure constraints research: intrapersonal constraints concern individual capability and willingness, interpersonal constraints concern coordination with travel companions, and structural constraints concern external conditions such as time, money, and accessibility [41]. Tourism research has likewise shown that perceptions of such constraints intervene substantively in travel decision processes [42]. PBC therefore captures the behavioral feasibility mechanism in the present model, functionally distinct from the behavioral evaluation mechanism captured by attitude.

Within TPB, stronger perceived control strengthens intention, because individuals are more likely to consider performing a behavior when they believe the necessary resources and opportunities are available and that salient constraints can be managed [18]. The effect of PBC in tourism research is not uniform, however, and this variability has been raised in recent critical assessments of the theory’s application to the field [25]. Two contrasting sets of findings illustrate this variability. In studies of Chinese visitors’ intention to visit World Cultural Heritage sites and to revisit a folk festival, PBC was the strongest predictor of intention, outperforming attitude and other tourism-specific constructs [21,22]. By contrast, in a recent short-video study of city tourism, PBC carried the smallest path coefficient, which the authors attributed to the declining economic constraints and increasing convenience of travel among Chinese residents [30]. Taken together, these findings suggest that the association between PBC and intention may vary with the resource demands of the focal travel activity. Recent evidence on young travelers is consistent with this, showing that higher perceived constraints correspond to weaker travel intentions [43].

SFT combines compressed itineraries, limited expenditure, and physically intensive activities [1,6,11]. These features may make time, budget, itinerary coordination, and physical capacity salient when potential travelers assess feasibility. They correspond to structural and intrapersonal constraints discussed in leisure and tourism research [41,42]. Research on hard-adventure tourism has also examined perceived control within an extended model of goal-directed behavior [44], providing a comparison with another resource-demanding activity. On this basis, individuals who consider SFT’s requirements manageable may report stronger intention, whereas those who perceive insufficient resources or capacity may report weaker intention even when they evaluate the activity favorably.

Taken together, the preceding arguments regarding constraint structure and evidence from tourism applications of TPB support the expectation of a positive association between perceived behavioral control and intention to engage in Special Forces-style tourism. Accordingly, the following hypothesis is proposed:

H3: Perceived behavioral control is positively associated with Chinese Gen Z university students’ intention to engage in Special Forces-style tourism.

### 2.5 Attitude and intention

Attitude toward SFT refers to an individual’s overall favorable or unfavorable evaluation of participating in this form of travel, including anticipated enjoyment, excitement, value, and meaningfulness [18,45]. It concerns whether participation is desirable, while PBC concerns feasibility and PCA and PCT concern evaluations of short-video content. TPB predicts that favorable evaluations of performing a behavior are associated with stronger intention to perform it [18]. This distinction provides the theoretical basis for examining attitude alongside the other antecedents in the present model.

Findings on attitude in tourism applications of TPB vary across settings. Some studies of Chinese or ethnically Chinese travelers found no significant relationship between attitude and visit or revisit intention [21,22,46]. More recent empirical studies reported positive associations between attitude and city travel intention in a short-video setting [30], and between attitude and hotel purchase intention [31]. These results provide direct empirical support for a positive association without implying that attitude is consistently the strongest predictor. Critical assessments of TPB in tourism also emphasize variation across research contexts [25]. The present study therefore defines attitude specifically as evaluation of participating in SFT, consistent with TPB’s behavior-specific definition [18].

SFT studies describe experiences of challenge, novelty, accomplishment, and personal meaning [1,6,11]. These features provide a contextual reason to consider attitude: potential participants may evaluate whether an intensive trip would be enjoyable and worthwhile. In line with TPB and its tourism applications [18,45], favorable evaluations of these anticipated experiences are expected to be associated with stronger participation intention.

Existing SFT research indicates that participants value the novelty, challenge, accomplishment, and experiential meaning associated with this travel form [1,4,11]. Qualitative studies further suggest that SFT can function as a means of self-expression and identity performance among young travelers [3,6,9,47]. These findings can be considered alongside broader tourism and consumer behavior research examining the roles of self-related evaluations, attitudes, and values in behavioral intentions [29,48]. In the SFT context, anticipated experiential and personal value provides a reason for individuals to evaluate participation favorably and to form stronger intentions to engage in it.

Taken together, TPB and the existing tourism literature support the expectation that Chinese Generation Z university students who evaluate participation in Special Forces-style tourism more favorably will report stronger intentions to engage in this form of travel. Accordingly, the following hypothesis is proposed:

H4: Attitude toward Special Forces-style tourism is positively associated with Chinese Gen Z university students’ intention to engage in Special Forces-style tourism.

### 2.6 Intention and self-reported behavior

Within the Theory of Planned Behavior, intention reflects an individual’s motivational readiness and willingness to perform a particular behavior and is regarded as one of the most proximal psychological determinants of behavioral engagement [18]. A positive relationship between intention and behavior has been widely documented in behavioral and tourism research, although strong intentions do not always correspond fully with subsequent behavioral engagement because actual opportunities, available resources, and situational constraints may affect whether a behavior is performed [19,49,50].

Both TPB and prior tourism research provide reasons to expect a positive association between these constructs. Intention is generally associated with tourism participation, although time constraints, transportation conditions, travel distance, financial resources, and other situational factors may weaken the degree of correspondence between intention and behavior [19,51]. This issue is particularly relevant to SFT because participation requires sufficient time, financial resources, physical capacity, and the ability to coordinate highly compressed itineraries [41,42]. Recent tourism research has likewise examined associations between intention and retrospective self-reported behavior while distinguishing such relationships from prospective or causal effects [52].

Because the present study uses a cross-sectional design, the temporal referents of intention and self-reported behavior are not prospectively aligned: intention captures current future-oriented plans and willingness, whereas the BEH items primarily reflect prior or habitual SFT-related travel patterns. Accordingly, H5 tests a cross-sectional statistical association, rather than the prospective sequence proposed by TPB in which intention measured at one point predicts subsequent behavior. The path should therefore not be interpreted as evidence that the measured intention caused the reported behavior. The following hypothesis is proposed:

H5: Intention to engage in Special Forces-style tourism is positively associated with self-reported SFT-related behavior among Chinese Generation Z university students.

#### 2.7 The moderating role of short-video platform usage intensity.

Short-Video Platform Usage Intensity (SVPUI) refers to routine engagement with short-video platforms for tourism information, travel inspiration, and related entertainment. Its items cover attention to likes, comments, and peer sharing, frequent viewing and searching for travel content, and the importance of platforms in everyday use [53,54]. It is therefore broader than time spent on a platform alone. In this study, SVPUI is examined as a moderator of the relationship between Intention and self-reported behavior.

The strength of the association between intention and self-reported behavior may vary according to individuals’ level of short-video platform engagement. Higher levels of platform use expose individuals more frequently to tourism-related information, repeated representations of comparable travel experiences, and ongoing travel-related interactions. Such repeated exposure may increase the cognitive salience of Special Forces-style tourism and provide continuing access to information about routes, schedules, costs, and travel arrangements, as well as examples of how other users organize similar trips [2,35,55,56]. Under these conditions, individuals with stronger SFT intentions may also report higher levels of related behavior because the platform environment provides continuing informational support and behavioral reference points for existing travel orientations. By contrast, individuals with lower levels of platform engagement encounter such information and interactions less frequently, and this association may therefore be weaker [57].

However, greater platform usage intensity does not necessarily strengthen the association between intention and self-reported behavior. As usage becomes more intensive, short-video consumption may become increasingly habitual or passive, such that repeated exposure to tourism content does not necessarily correspond to higher levels of behavioral engagement [53,54]. Highly homogenized exposure generated by algorithmic recommendation may also reduce the novelty of similar travel content and thereby weaken its relevance to specific tourism judgments and behavior [58]. From a theoretical perspective, therefore, higher SVPUI may either strengthen this association or weaken it through habitual consumption, passive engagement, or content homogenization.

Given these competing mechanisms, the present study does not prespecify the direction of the moderating effect. Instead, it empirically examines whether SVPUI changes the strength of the intention–BEH relationship. Accordingly, the following hypothesis is proposed:

H6: Short-video platform usage intensity moderates the relationship between intention to engage in Special Forces-style tourism and self-reported SFT-related behavior among Chinese Gen Z university students.

Based on the above theoretical analysis and hypothesis development, the proposed research framework of this study is presented in Fig 1.

**Fig 1:**
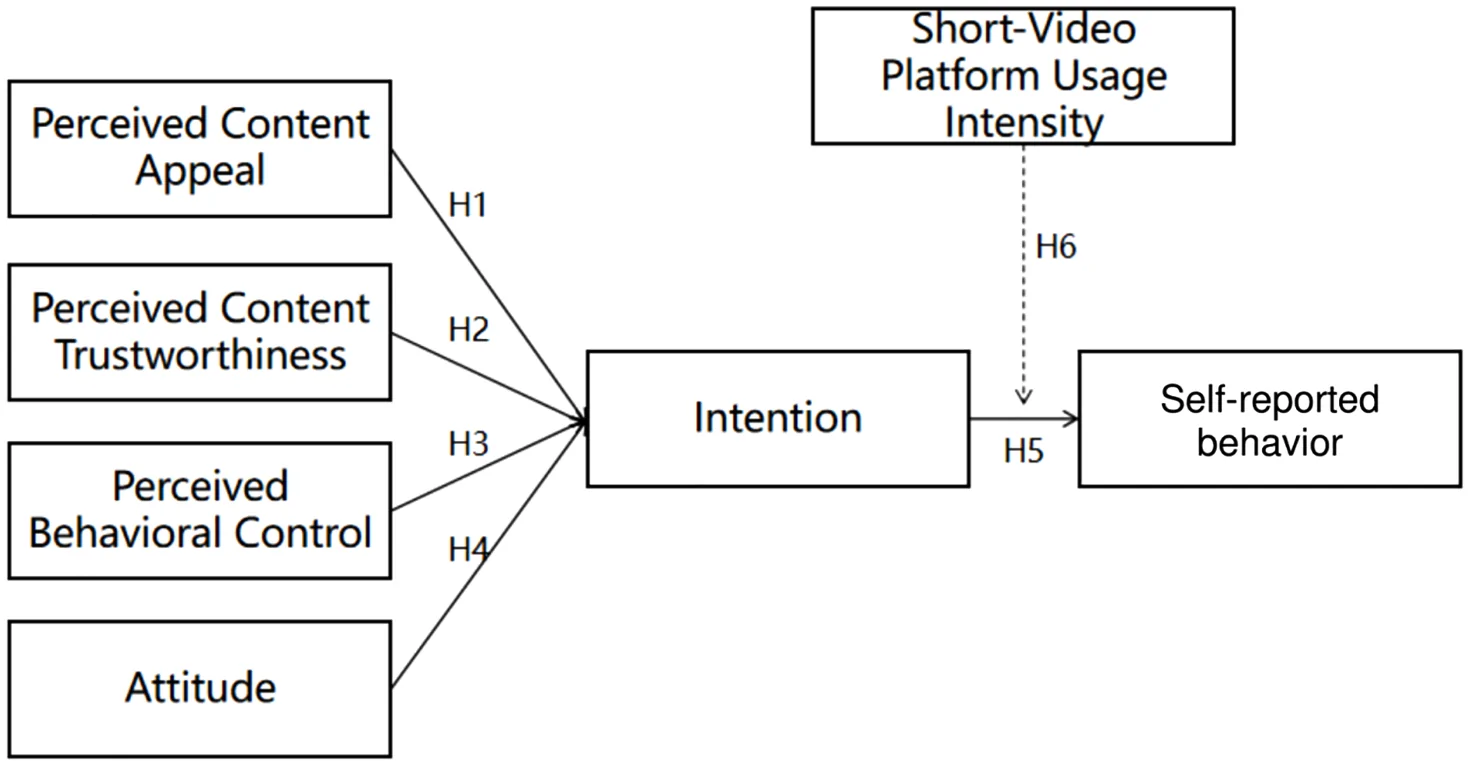
Proposed research framework.

## 3. Research design and methodology

### 3.1 Sampling and survey administration

This study employed a cross-sectional online survey to examine relationships among short-video content perceptions, TPB-related constructs, short-video platform usage intensity, intention to engage in Special Forces-style tourism (SFT), and self-reported SFT-related behavior among Chinese Generation Z university students.

A province-based, multi-site sampling strategy was used in Hunan and Guangdong Provinces to broaden the geographic and institutional coverage of the sample. Data were collected from students at six universities: Hunan University, Central South University, and Changsha Normal University in Hunan Province, and Sun Yat-sen University, Jinan University, and Shenzhen University in Guangdong Province. The inclusion of multiple universities across the two provinces was intended to reduce reliance on a single institutional setting and to capture students from different university environments. The survey was specifically advertised and distributed to Chinese Generation Z university students enrolled at mainland Chinese universities, consistent with the predefined target population of the study. University students were selected because they constitute an important participant group in SFT and their budget, time, and digital-media-use characteristics correspond closely to this travel form [12]. The study therefore focuses specifically on the university-student segment of Chinese Generation Z and does not assume that these participants represent the entire Generation Z population.

The Chinese-language questionnaire was administered online through Wenjuanxing from September 1 to December 30, 2025. After contacts were established at each participating university, the online questionnaire link was circulated among students. Students voluntarily shared the link with peers at their universities. Before entering the substantive questionnaire, participants viewed the study information and informed-consent statement, which explained the purpose of the study, eligibility requirements, voluntary nature of participation, anonymity, and intended use of the data. Participation was restricted to individuals aged 18 years or older, and respondents could proceed only after providing electronic informed consent. Two subsequent screening questions confirmed that respondents were currently enrolled at a mainland Chinese university and had previously encountered SFT-related short-video content. Responses that failed the eligibility criteria or contained substantial missing data were excluded. A total of 700 questionnaire responses were received, of which 526 were retained as usable responses after screening.

### 3.2 Questionnaire development and pilot study

The questionnaire included two eligibility screening questions, background questions on demographics, travel experience, and short-video use, and 28 measurement items covering seven constructs—Perceived Content Appeal (PCA), Perceived Content Trustworthiness (PCT), Attitude, Perceived Behavioral Control (PBC), Intention, Self-reported behavior (BEH), and Short-Video Platform Usage Intensity (SVPUI). All construct items used a five-point Likert scale. Table 1 reports the seven background characteristics relevant to the study. Detailed measurement items and their sources are provided in S1 Appendix.

**Table 1:** Demographic and travel-related characteristics of respondents. Frequencies (n=526).

Items	Category	Frequency	Percent (%)	Cumulative Percent (%)
Gender	Male	230	43.7	43.7
	Female	296	56.3	100
Current education level	Undergraduate	354	67.3	67.3
	Master’s student	147	27.9	95.2
	Doctoral student	5	1.0	96.2
	Other	20	3.8	100
Daily time spent on short-video platforms	0–30 minutes	17	3.2	3.2
	30–60 minutes	61	11.6	14.8
	1–2 hours	119	22.6	37.5
	More than 2 hours	329	62.5	100
Posted tourism-related short-videos on social media	Yes	386	73.4	73.4
	No	140	26.6	100
Traveled across cities or provinces in the past year	Yes	467	88.8	88.8
	No	59	11.2	100
Experience of “Special Forces-style tourism”	Yes, at least once	387	73.6	73.6
	No, but want to try	102	19.4	93
	No, and do not want to try	37	7	100
Monthly disposable living expenses (RMB)	Less than 1,000	35	6.7	6.7
	1,000–2,000	175	33.3	39.9
	2,000–3,000	278	52.9	92.8
	3,000–5,000	25	4.8	97.5
	More than 5,000	13	2.5	100
Total		526	100	100

Items were adapted from published measures and construct definitions, translated into Chinese, and contextualized for SFT and short-video use. Five tourism and consumer-behavior scholars and two short-video practitioners reviewed the items for clarity and contextual fit. Where more than one publication informed a construct, sources were combined to cover complementary facets of the same construct. For example, PCA includes both affective appeal and informational usefulness, whereas PBC includes perceived capability and resource availability. Item selection and review focused on conceptual consistency across these facets. S1 Appendix identifies the sources informing each construct.

An online pilot study conducted from July 1 to August 1, 2025 yielded 112 valid responses. The pilot was not limited to readability testing; it also provided a preliminary assessment using Cronbach’s α, KMO, Bartlett’s test, and exploratory factor analysis. A minimum of approximately 100 respondents for exploratory factor analysis is discussed by Rattray and Jones [59]; this supported using a larger pilot for preliminary psychometric assessment. The pilot produced Cronbach’s α values of 0.820–0.896, a KMO of 0.872, a significant Bartlett’s test (χ² = 1971.61, df = 378, p < 0.001), and seven factors explaining 73.77% of total variance.

### 3.3 PLS-SEM and common method bias

SmartPLS was used for the PLS-SEM analyses because the model contains multiple latent constructs, a moderation effect, and an explanatory-predictive focus [60]. The measurement model was assessed using indicator loadings, Cronbach’s α, composite reliability, AVE, and discriminant validity; the structural model was assessed using collinearity, path coefficients, and R^2^. Stone–Geisser Q^2^ was obtained from the blindfolding cross-validated redundancy output. Statistical significance was tested using nonparametric bootstrapping with 5,000 subsamples. The moderating effect of SVPUI was assessed through the Intention × SVPUI interaction term, estimated with its lower-order terms, and interpreted using a moderation plot. H6 concerns the interaction term.

Because all focal constructs were measured from the same respondents at one time point, common method bias (CMB) was assessed using two complementary procedures. First, Harman’s single-factor test entered all 28 measurement items into an unrotated factor analysis. Second, following Kock [61], full-collinearity VIFs were assessed for the seven focal constructs using 3.3 as the conservative threshold. The results are reported in the Results section.

### 3.4 fsQCA procedure

fsQCA 3.0 was used to complement PLS-SEM by examining conjunctural relationships, equifinality, and configurational asymmetry [24,62]. The same 526 cases were analyzed, with PCA, PCT, Attitude, PBC, Intention, and SVPUI as antecedent conditions and self-reported behavior as the outcome. Standardized latent variable scores generated by SmartPLS 4 were exported as inputs for the fsQCA analysis. This use of model-derived construct scores follows methodological guidance on combining PLS-SEM and fsQCA [24] and the approach illustrated by D. Wang et al. [63].

The latent variable scores were calibrated into fuzzy sets using the direct calibration method. Because no theoretically established external thresholds were available for the focal constructs, the 5th, 50th, and 95th percentiles were used as the anchors for full non-membership, the crossover point, and full membership, respectively [62,64]. These distribution-based anchors define relative set membership within the sample. Fixed cutoffs on the original Likert scale would not map directly onto standardized latent variable scores. No calibrated membership score was exactly equal to 0.500, so no further adjustment was required.

Necessity analyses were conducted separately for high and low self-reported behavior, using a consistency threshold of 0.90. Truth tables were then constructed for both outcomes. Given the sample size of 526, a frequency cutoff of 3 was applied, together with a raw consistency threshold of 0.80 and a proportional reduction in inconsistency (PRI) threshold of 0.70.

Intermediate solutions were used to interpret sufficient configurations, while parsimonious solutions were used to distinguish core from peripheral conditions. Conditions appearing in both solution types were classified as core, whereas those appearing only in the intermediate solution were classified as peripheral. Raw coverage, unique coverage, consistency, overall solution coverage, and overall solution consistency were reported for the final configurations.

### 3.5 Ethical approval and informed consent

The study was reviewed and approved by the Faculty of Economics and Management, Changsha Normal University (approval number: CSNU-FEM-2025–001; approval date: 5 June 2025). The study used an anonymous, non-interventional online questionnaire restricted to university students aged 18 years or older. Before answering the substantive questionnaire, participants received information about the study purpose, voluntary participation, anonymity, data use, and withdrawal rights and were required to confirm their electronic informed consent. Participants could discontinue the questionnaire at any time. No sensitive personal information unrelated to the research objectives was collected.

## 4. Results

### 4.1 Sample characteristics

The demographic profile of the respondents is summarized in Table 1. Among the 526 valid participants, females accounted for 56.3%, while males comprised 43.7%. Most respondents were undergraduates (67.3%), followed by master’s students (27.9%) and a small proportion of doctoral students (1%) and others (3.8%). A majority (62.5%) reported spending more than two hours daily on short-video platforms, and 73.4% had posted tourism-related short videos on social media. Regarding travel experience, 88.8% had engaged in cross-city or cross-province travel in the past year, and 73.6% had experienced “Special Forces-style tourism” at least once, with an additional 19.4% expressing a desire to try it. The majority (52.9%) reported monthly disposable living expenses of RMB 2,000–3,000. Overall, these descriptive statistics indicate that the study sample was characterized by relatively high levels of short-video platform use and substantial recent travel experience.

### 4.2 Measurement analysis

Table 2 presents the results of the reliability and convergent validity assessment for all latent constructs. ATT, INT, and BEH denote attitude, intention, and self-reported behavior, respectively; the BEH1–BEH4 item codes are retained throughout the tables and S1 Appendix. All indicator loadings ranged from 0.815 to 0.883, exceeding the recommended threshold of 0.70 [60], indicating high indicator reliability. Cronbach’s α values ranged from 0.863 to 0.884, while composite reliability (CR) values ranged from 0.907 to 0.920, both above the recommended minimum of 0.70 [65]. Average variance extracted (AVE) values ranged from 0.708 to 0.742, exceeding the 0.50 benchmark [66] and supporting convergent validity.

**Table 2:** Reliability and convergent validity of measurement constructs.

Construct	Item	Loading	Cronbach’s Alpha (α)	Composite Reliability	AVE
Self-reported behavior	BEH1	0.815	0.871	0.912	0.721
	BEH2	0.862			
	BEH3	0.865			
	BEH4	0.854			
Attitude	ATT1	0.836	0.865	0.908	0.711
	ATT2	0.836			
	ATT3	0.843			
	ATT4	0.859			
Intention	INT1	0.852	0.865	0.908	0.711
	INT2	0.834			
	INT3	0.847			
	INT4	0.839			
PBC	PBC1	0.853	0.874	0.913	0.725
	PBC2	0.852			
	PBC3	0.856			
	PBC4	0.844			
PCA	PCA1	0.857	0.884	0.920	0.742
	PCA2	0.869			
	PCA3	0.883			
	PCA4	0.835			
PCT	PCT1	0.858	0.863	0.907	0.708
	PCT2	0.827			
	PCT3	0.853			
	PCT4	0.828			
SVPUI	SVPUI1	0.836	0.868	0.910	0.716
	SVPUI2	0.858			
	SVPUI3	0.844			
	SVPUI4	0.848			

Table 3 reports the discriminant validity results based on the Fornell–Larcker criterion. For each construct, the square root of AVE (diagonal elements in bold) exceeded all corresponding inter-construct correlations, indicating that each construct shares more variance with its indicators than with the other constructs under this criterion [66]. These results collectively demonstrate that the measurement model meets the criteria for reliability, convergent validity, and discriminant validity, providing a solid foundation for subsequent structural model analysis.

**Table 3:** Discriminant validity assessment.

	Self-reported behavior	Attitude	Intention	PBC	PCA	PCT	SVPUI
Self-reported behavior	**0.849**						
Attitude	0.371	**0.843**					
Intention	0.449	0.545	**0.843**				
PBC	0.394	0.453	0.506	**0.851**			
PCA	0.422	0.439	0.455	0.402	**0.861**		
PCT	0.503	0.476	0.493	0.497	0.479	**0.842**	
SVPUI	0.336	0.446	0.44	0.474	0.39	0.486	**0.846**

Collinearity was first assessed for the structural model using inner VIF values. These values ranged from 1.009 to 1.608, indicating no problematic multicollinearity among the predictor constructs. Because all focal constructs were self-reported by the same respondents at the same point in time, common method bias (CMB) was additionally assessed using two complementary procedures. Harman’s single-factor test showed that the first unrotated factor accounted for 38.04% of the total variance across the 28 measurement items, below the commonly used 50% benchmark. In addition, following Kock [61], full-collinearity VIFs were calculated for all seven constructs: Attitude = 1.671, Intention = 1.804, self-reported behavior = 1.497, PBC = 1.642, PCA = 1.523, PCT = 1.838, and SVPUI = 1.542. All values were below the conservative threshold of 3.3.

### 4.3 Evaluation of the structural model

The structural model results are presented in Fig 2, and the hypothesis-testing results are summarized in Table 4. The R^2^ values for Intention and self-reported behavior were 0.429 and 0.263, respectively, indicating that the predictors included in the model explained 42.9% of the variance in Intention and 26.3% of the variance in BEH. The comparatively lower R^2^ for this outcome also indicates that a substantial proportion of its variance remains unexplained by the variables included in the present model. The Stone–Geisser Q^2^ values based on cross-validated redundancy were 0.300 for Intention and 0.179 for BEH. Both values were greater than zero, indicating predictive relevance for the two endogenous constructs [60].

**Table 4:** Structural model results and hypothesis testing.

Hypothesis	Path	β	t-value	p-value	Decision
H1	PCA → **Intention**	0.155	3.791	<0.001	**Supported**
H2	PCT → **Intention**	0.165	3.841	<0.001	**Supported**
H3	PBC → **Intention**	0.228	5.253	<0.001	**Supported**
H4	**Attitude** → **Intention**	0.296	6.893	<0.001	**Supported**
H5	**Intention** → **Self-reported behavior**	0.355	7.937	<0.001	**Supported**
H6	SVPUI × **Intention** → **Self-reported behavior**	0.206	4.680	<0.001	**Supported**

**Fig 2:**
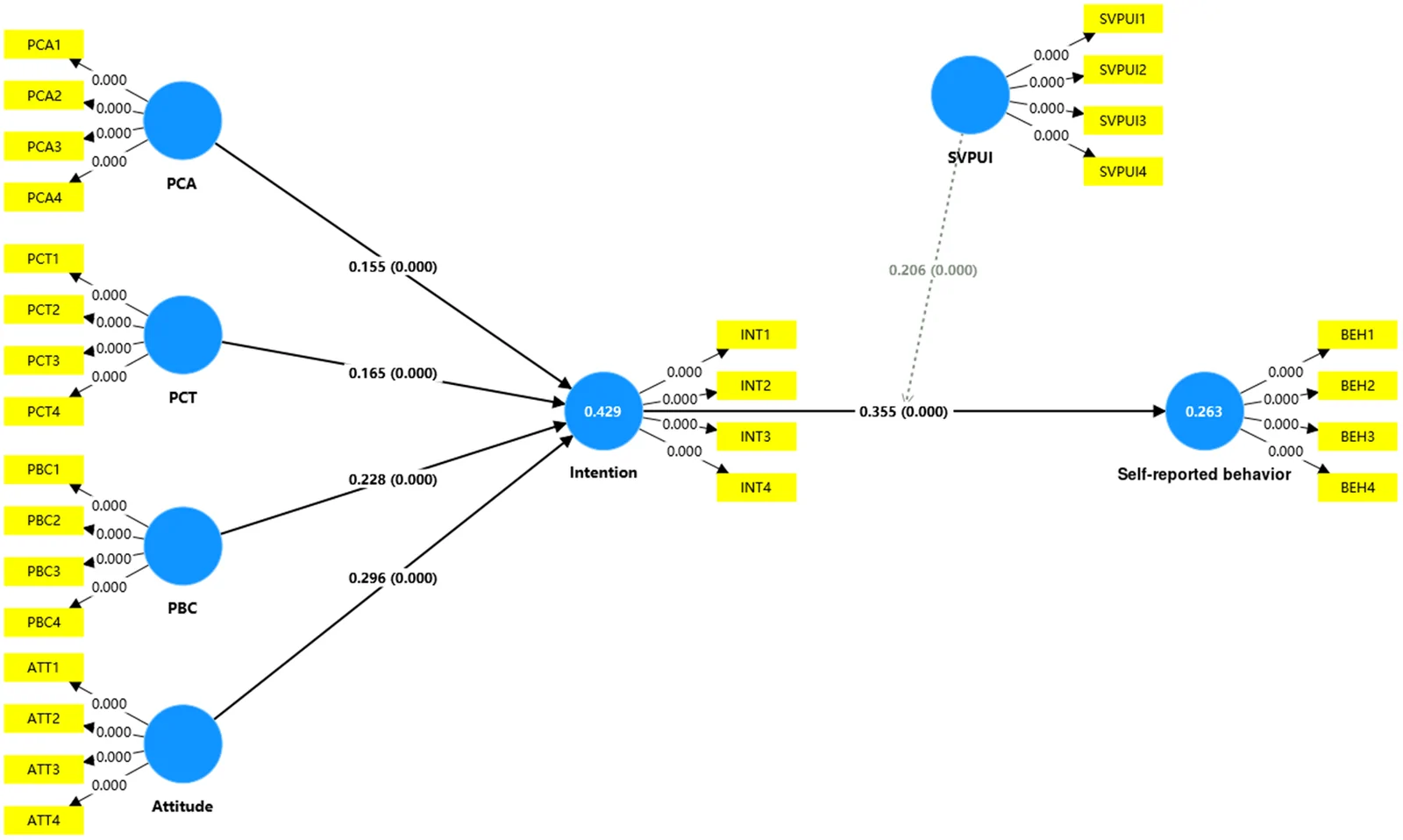
Structural model results. Path labels show coefficients with p-values in parentheses; values displayed as 0.000 indicate p < 0.001. Values inside endogenous constructs are R². The figure displays the hypothesized relationships.

The path estimates showed that PCA (β = 0.155, t = 3.791, p < 0.001), PCT (β = 0.165, t = 3.841, p < 0.001), PBC (β = 0.228, t = 5.253, p < 0.001), and Attitude (β = 0.296, t = 6.893, p < 0.001) were all positively and significantly associated with Intention. Thus, H1–H4 were supported. Among the four predictors of Intention, Attitude exhibited the largest standardized path coefficient. Intention was positively and significantly associated with self-reported behavior (β = 0.355, t = 7.937, p < 0.001), supporting H5.

The interaction between SVPUI and Intention was positively and significantly associated with self-reported behavior (β = 0.206, t = 4.680, p < 0.001), supporting H6. As illustrated in Fig 3, the association between Intention and BEH was relatively weaker at a low level of SVPUI (−1 SD), stronger at the mean level of SVPUI, and strongest at a high level of SVPUI (+1 SD). The fitted lines cross at a relatively low level of Intention, so higher SVPUI does not correspond to higher fitted BEH scores throughout the plotted range. The positive interaction concerns the difference in slopes across SVPUI levels; the figure does not provide separate significance tests for the three conditional slopes.

**Fig 3:**
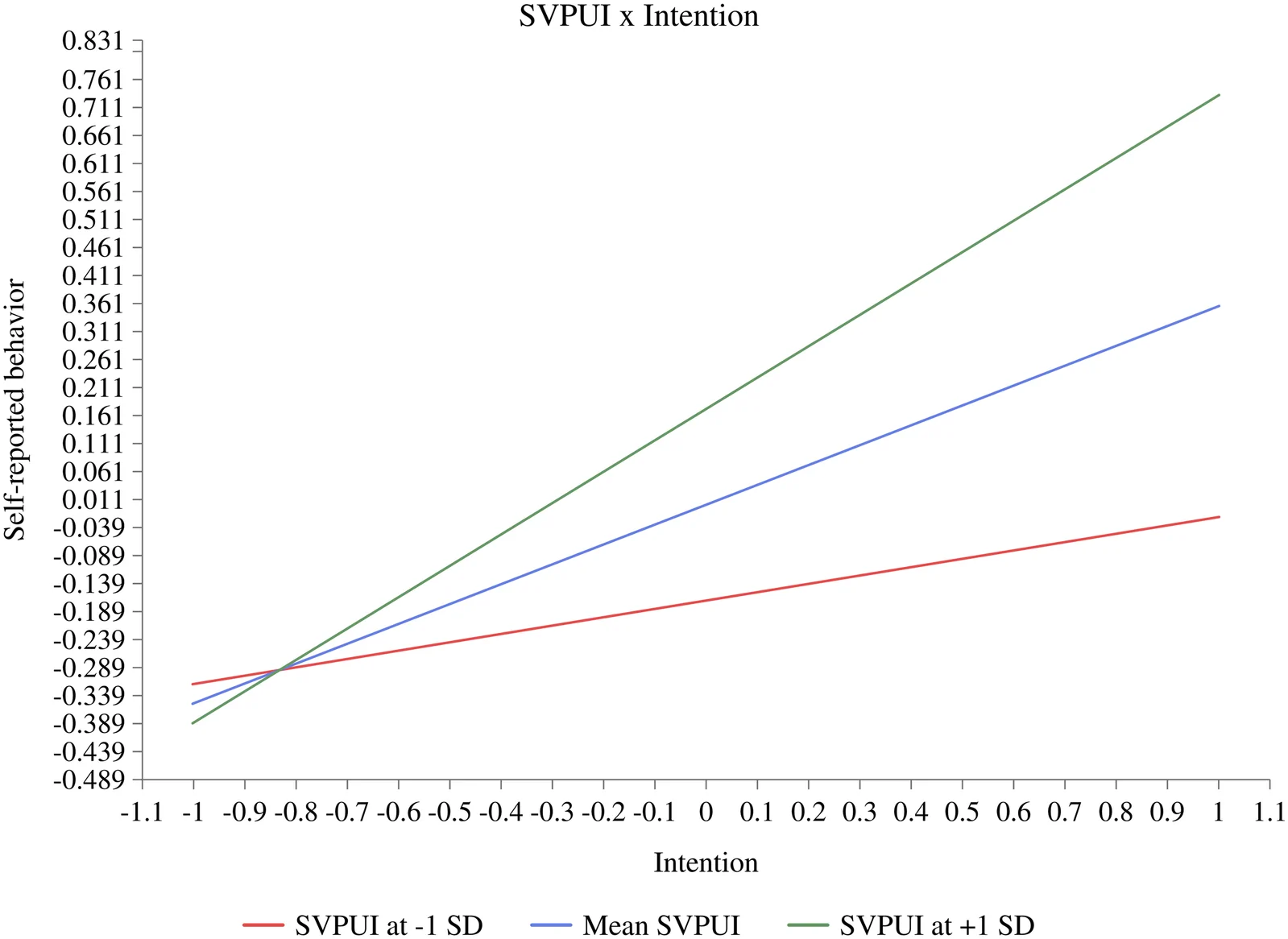
Interaction effect of short-video platform usage intensity on the relationship between intention and self-reported behavior. Lines represent SVPUI at −1 SD, its mean, and +1 SD. Axes use standardized construct scores.

### 4.4 Research results from fsQCA

To complement the PLS-SEM analysis, fsQCA was conducted using the standardized latent variable scores generated by SmartPLS 4. The analysis examined configurations of Attitude, Intention, PCA, PCT, PBC, and SVPUI associated with high and low levels of self-reported behavior. The following subsections report the calibration results, necessity analysis, and sufficient configurations.

#### 4.4.1. Calibration.

Following the calibration procedure described in Section 3.4, the standardized latent variable scores obtained from the PLS-SEM analysis were used as the inputs for fsQCA. The 5th, 50th, and 95th percentiles of each construct were used as the empirical anchors for full non-membership, the crossover point, and full membership, respectively. Table 5 presents the resulting calibration anchors. Because none of the calibrated membership scores was exactly equal to 0.500 at the precision used in the analysis, no additional adjustment of crossover scores was required.

**Table 5:** Calibration anchors for each variable.

Anchor	ATT	INT	BEH	PCA	PCT	PBC	SVPUI
Full non-membership (5th percentile)	−1.507784	−1.715293	−1.722260	−1.674220	−1.456064	−1.685244	−1.714191
Crossover point (50th percentile)	0.098086	0.087582	0.095909	0.080568	−0.001564	0.063831	0.077704
Full membership (95th percentile)	1.413390	1.391414	1.673270	1.593948	1.439105	1.606070	1.387602

#### 4.4.2 Necessity analysis.

Before conducting the sufficiency analysis, necessity analyses were performed separately for high and low self-reported behavior. Table 6 reports the consistency and coverage values for each antecedent condition and its negation. Following the criterion specified in Section 3.4, a consistency threshold of 0.90 was used to identify necessary conditions.

**Table 6:** Necessity analysis for high and low self-reported behavior.

Condition	High self-reported behavior Consistency	High self-reported behavior Coverage	Low self-reported behavior Consistency	Low self-reported behavior Coverage
Attitude	0.725	0.717	0.551	0.558
~Attitude	0.553	0.547	0.721	0.728
Intention	0.753	0.731	0.552	0.548
~Intention	0.534	0.538	0.729	0.751
PCA	0.746	0.739	0.552	0.559
~PCA	0.555	0.548	0.742	0.750
PCT	0.762	0.747	0.521	0.523
~PCT	0.513	0.512	0.748	0.763
PBC	0.731	0.726	0.558	0.567
~PBC	0.564	0.555	0.730	0.736
SVPUI	0.737	0.711	0.570	0.562
~SVPUI	0.546	0.554	0.707	0.734

Note: “~” denotes the absence (negation) of a condition. A consistency threshold of 0.90 was used to identify necessary conditions.

None of the conditions or their negations reached the 0.90 threshold for either high or low self-reported behavior. Therefore, no single antecedent condition was necessary for either outcome. For the high-outcome set, PCT exhibited the highest necessity consistency (0.762), followed by Intention (0.753) and PCA (0.746). For the low-outcome set, the absence of PCT (~PCT) showed the highest consistency (0.748), followed by the absence of PCA (~PCA; 0.742) and the absence of PBC (~PBC; 0.730). However, all consistency values remained well below the 0.90 threshold for necessity, indicating that no single condition constituted a necessary condition for either outcome.

#### 4.4.3 Configurations associated with high and low self-reported behavior.

Using the analytical criteria specified in Section 3.4, truth-table rows were retained when they met a frequency cutoff of 3, a raw consistency threshold of at least 0.80, and a PRI consistency threshold of at least 0.70. After applying these joint criteria, the lowest raw consistency among the retained truth-table rows was 0.920 for high BEH and 0.936 for low BEH. The intermediate solutions were used as the primary basis for interpreting the sufficient configurations, while the parsimonious solutions were used to distinguish core from peripheral conditions.

Two sufficient configurations were identified for high self-reported behavior. Intention, PBC, PCT, and SVPUI constituted core conditions in both configurations. HB1 additionally contained Attitude as a peripheral condition, whereas HB2 contained PCA as a peripheral condition. Their raw coverage values were 0.483 and 0.487, respectively, and their consistencies were 0.903 and 0.907. The overall solution coverage was 0.519 and the solution consistency was 0.893. The relatively low unique coverage values of 0.032 and 0.036 indicate substantial overlap between the cases covered by the two configurations.

Eight sufficient configurations were identified for low self-reported behavior. The absence of PCT (~PCT) occurred in six of the eight configurations, indicating that low PCT was a particularly recurrent element in these pathways. The remaining conditions varied substantially across the configurations, including configurations in which relatively high levels of Intention, Attitude, or PBC coexisted with the absence of other antecedents. Raw coverage ranged from 0.300 to 0.530, while consistency ranged from 0.877 to 0.948. The overall solution coverage was 0.686 and the solution consistency was 0.839.

The solutions for high and low self-reported behavior were not simple inversions of one another. This configurational asymmetry indicates that low-outcome combinations cannot be inferred merely by reversing the conditions in high-outcome combinations (Table 7).

**Table 7:** Configurations associated with high and low self-reported behavior.

Conditions	HB1	HB2	LB1	LB2	LB3	LB4	LB5	LB6	LB7	LB8
Attitude	•					⊗	⊗	⊗	●	⊗
Intention	●	●	⊗		⊗		⊗	•	⊗	⊗
PCA		•		⊗	⊗			⊗	⊗	⊗
PCT	●	●	⊗	⊗	⊗	⊗	⊗	⊗		
PBC	●	●	⊗	⊗		⊗			⊗	●
SVPUI	●	●		⊗	⊗	⊗	⊗		⊗	⊗
**Raw coverage**	.483	.487	.530	.460	.458	.457	.459	.324	.300	.306
**Unique coverage**	.032	.036	.068	.015	.007	.010	.007	.022	.013	.011
**Consistency**	.903	.907	.877	.918	.908	.900	.894	.924	.948	.942
**Solution coverage**	.519	.519	.686	.686	.686	.686	.686	.686	.686	.686
**Solution consistency**	.893	.893	.839	.839	.839	.839	.839	.839	.839	.839

Note: ● = core condition present; • = peripheral condition present; ⊗ = core condition absent; ⊖ = peripheral condition absent; blank cells indicate that either presence or absence of the condition is compatible with the configuration (a “do not care” condition). HB1–HB2 denote configurations associated with high self-reported behavior, whereas LB1–LB8 denote configurations associated with low self-reported behavior. Core and peripheral conditions were identified by comparing the intermediate solutions with their corresponding parsimonious prime implicants.

## 5. Discussion

This study addressed four research questions concerning the relationships among short-video content perceptions, TPB-related constructs, platform usage intensity, Intention, and self-reported SFT-related behavior among Chinese Generation Z university students. The PLS-SEM results showed that PCA, PCT, Attitude, and PBC were all positively associated with Intention, which was in turn positively associated with BEH. SVPUI also moderated this association, which became stronger at higher levels of platform usage intensity. The subsequent fsQCA analysis further showed that high and low levels of this outcome were associated with different combinations of conditions, indicating that SFT-related behavior involves not only average net associations among variables but also configurational patterns. The discussion first considers the structural relationships identified by PLS-SEM before turning to the configurational findings.

### 5.1 Linear structural insights from PLS-SEM

First, PCA and PCT were both positively associated with Intention and had similar estimated standardized coefficients. Tourism short-video research links visual presentation, emotional engagement, and informational usefulness with audiences’ interest and travel intention [16,17,33]. Digital tourism studies also emphasize authenticity, accuracy, and reliability in judgments about online travel information [14,15,36]. In SFT, appealing content may stimulate interest, while trustworthy content may help potential travelers judge whether information about routes, timing, budgets, and itineraries provides a realistic basis for planning.

Among the four antecedents of Intention, Attitude showed the largest standardized association (β = 0.296), followed by PBC (β = 0.228). This pattern is broadly consistent with the central TPB logic that behavioral evaluation and perceived control are important components of intention formation, while also reflecting characteristics specific to SFT. Attitude captures whether individuals evaluate participation in SFT as enjoyable, valuable, and worth attempting. Previous research has associated SFT among young travelers with challenge, novelty, self-expression, and a sense of travel accomplishment [1,6,11]. University students who evaluate this high-intensity travel form more favorably may therefore be more likely to report stronger intentions to participate.

The PBC finding is also meaningful. Tourism applications of TPB have not produced a uniform pattern for PBC, and its relative explanatory role appears to depend partly on the extent to which the focal behavior is constrained by time, resources, and individual capability [21,22,30]. SFT places clear demands on time management, budget control, itinerary coordination, and physical capacity. Judgments about whether one possesses the resources and capabilities required to complete a compressed and intensive trip may therefore be particularly salient. This interpretation is consistent with leisure-constraint and tourism decision-making research, which emphasizes the roles of time, financial resources, individual capacity, and actual opportunities in shaping travel choices [41,42]. Potential participants may therefore evaluate not only whether SFT is desirable, but also whether it appears personally feasible.

Second, Intention was positively associated with self-reported behavior, consistent with prior tourism and behavioral research [18,19,50]. The model explained 42.9% of the variance in Intention. This falls between the commonly cited PLS-SEM benchmarks of 0.25 and 0.50 for weak and moderate explanatory power, respectively; such benchmarks are contextual guidelines rather than universal standards for tourism and social research [60]. The model thus explains a meaningful but incomplete share of variation in Intention. The R^2^ of 0.263 for BEH indicates more limited explanatory power and leaves substantial variance unaccounted for. Unmeasured resource availability and situational constraints are plausible additional correlates, rather than explanations tested by this study. The difference between the two R^2^ values does not demonstrate that the model explains an intention–behavior gap. These boundaries are considered further in Section 5.5.

Finally, the moderating effect of SVPUI addresses RQ3: the positive association between Intention and self-reported behavior was stronger at higher levels of platform usage intensity. One plausible explanation is that frequent platform engagement provides continuing access to information about routes, budgets, transportation arrangements, and comparable travel experiences [2,35,55,56]. Such information has particular contextual relevance to SFT, which requires participants to coordinate transport connections, the sequence of attraction visits, and intensive activities within limited time and budgets. Continued exposure may help users integrate separate pieces of destination information into comparable travel plans and assess their suitability against their own resources and previous travel experiences. Repeated encounters with route examples, cost information, and shared experiences may also keep the practical requirements of SFT salient and readily accessible, offering an explanation for the closer correspondence between these constructs at higher levels of platform usage intensity.

This finding also complements the relationships of PCA and PCT with Intention. PCA and PCT capture users’ evaluations of the appeal and trustworthiness of short-video content, respectively, while SVPUI reflects their sustained engagement with the relevant platform environment. Content evaluation and platform engagement therefore have distinct explanatory roles in the model: the former is associated with participation intention, while the latter corresponds to differences in the strength of the intention–BEH association. The moderation finding highlights the relevance of the digital-media environment in which individuals repeatedly encounter and use tourism information when interpreting this relationship in SFT.

### 5.2 Configurational insights from fsQCA

The fsQCA results indicate that self-reported SFT-related behavior exhibits configurational characteristics. No single condition met the necessity threshold. Both configurations for high BEH contained Intention, PCT, PBC, and SVPUI as core conditions, with Attitude and PCA appearing as alternative peripheral conditions. High levels of reported SFT-related travel patterns were therefore associated with combinations of current motivational readiness, trustworthy content perceptions, perceived feasibility, and platform engagement. The two configurations suggest alternative supplementary roles for evaluation of the travel activity and appeal of the content depicting it. These are configurational associations in the present sample, consistent with conjunction and equifinality.

By contrast, low self-reported behavior is associated with a larger and more heterogeneous set of configurations. The absence of PCT appears repeatedly across most of these configurations, suggesting that insufficient content trustworthiness may constitute an important configurational factor associated with lower levels of this outcome. Prior digital tourism research has likewise shown that the perceived authenticity, accuracy, and reliability of online tourism information influence whether users are willing to rely on such information for travel judgment and planning [14,15,36]. However, the absence of PCT is not the only route to low BEH. Some such configurations still include favorable Attitude or relatively high PBC, indicating that a single favorable condition can coexist with low BEH. The solutions for high and low outcome levels therefore exhibit clear configurational asymmetry rather than functioning as simple opposites.

The two methods address different analytical questions. PLS-SEM estimates average associations with Intention and self-reported behavior, while fsQCA examines combinations associated specifically with high and low levels of this outcome. Attitude’s comparatively large coefficient for Intention therefore cannot be directly ranked against its peripheral status in a configuration for BEH: the outcomes and analytical criteria differ. Considered together, the analyses show how associations with future-oriented Intention can coexist with varied combinations related to reported travel patterns [24,63].

### 5.3 Theoretical implications

First, the study distinguishes evaluations of short-video content from evaluations of participating in SFT. Attitude and PBC capture the desirability and perceived feasibility of participation, while PCA and PCT capture the appeal, usefulness, and trustworthiness of the content representing it [14,16,17]. Within the specified model, constructs at both levels were positively associated with intention. These findings support considering content evaluations alongside selected TPB constructs when examining SFT intention in short-video settings. Because subjective norm was not measured and alternative model specifications were not compared, the findings do not establish the sufficiency of the modified framework or the irrelevance of normative influence.

Second, the study provides a more contextualized understanding of the intention–self-reported behavior relationship in digitally mediated tourism. TPB treats intention as a proximal psychological antecedent of behavior, although the strength of correspondence between intention and behavioral engagement may vary across contexts [18,19,50]. The present study found a significant positive association between Intention and self-reported SFT-related behavior, and this association became stronger as SVPUI increased. This suggests that platform usage intensity can be conceptualized as a digital-media context under which the strength of this association varies. More intensive platform engagement provides more frequent exposure to information concerning routes, budgets, transportation arrangements, and the experiences of other travelers, which may be associated with a closer correspondence between existing travel intentions and related behavioral patterns [2,35,55]. The finding concerns differences in an observed association across levels of platform engagement and does not demonstrate that platform use converts intention into subsequent participation.

Third, the combined analysis contributes a configurational account of self-reported SFT-related behavior. Both configurations for high BEH shared Intention, PCT, PBC, and SVPUI, while Attitude and PCA appeared as alternative peripheral conditions. Low BEH involved a more diverse set of configurations, with ~PCT appearing in six of eight pathways. Thus, the recurrence of content trustworthiness across configurations adds information beyond its association with Intention in PLS-SEM. This comparison concerns complementary findings for different analytical targets, rather than a ranking of path coefficients against core status. Neither PCT nor ~PCT met the necessity threshold, so content trustworthiness should be understood as a recurrent component of combinations, rather than as a universal prerequisite for this outcome.

### 5.4 Practical implications

The findings first suggest that SFT-related short-video communication should emphasize not only visual appeal and emotional stimulation but also credibility and practical feasibility. Platforms, content creators, destination marketing organizations, and tourism service providers can accompany engaging short-video narratives with clearer information on actual transportation time, ticket and accommodation costs, attraction opening hours, reservation requirements, walking distances, luggage-storage options, and first and last public-transport services. For highly compressed itineraries, content should also communicate realistic travel durations and physical demands rather than allowing editing to imply that large numbers of attractions can be completed easily within very short periods. Destinations may further provide realistic one- or two-day routes, expected budget ranges, and itineraries with different levels of intensity so that potential travelers can assess whether a proposed trip is compatible with their own time, financial resources, and physical readiness. Such information can simultaneously enhance content credibility and reduce uncertainty in feasibility judgments.

Second, the moderating role of SVPUI indicates that tourism communication should accommodate different levels of digital-media engagement. For young travelers who use short-video platforms intensively, short videos can function not only as sources of inspiration but also as gateways to practical planning resources. Itinerary videos, for example, can be linked to maps, transportation information, reservation systems, budget guidance, and official destination resources, making relevant planning information easier to access for users already interested in SFT. Destinations should not, however, assume that all young travelers depend equally on short-video platforms. Consistent route, transportation, and service information should also be available through official destination websites, online travel platforms, and other digital channels commonly used by university students. The managerial implication is to make reliable and actionable information accessible to travelers with different patterns of media engagement.

Third, the fsQCA findings caution against single-factor or one-size-fits-all management strategies. High self-reported behavior was associated with conjunctions of favorable conditions, and no single condition met the necessity threshold for low self-reported behavior. Managers may therefore benefit from combining common foundational support with responses targeted at different barriers. Credible information, clear itinerary design, budgeting and transportation support, and accessible digital planning resources can provide a common foundation. Beyond this, these differing configurations call for different responses. Where credibility is weak, greater transparency concerning costs, schedules, and itineraries may be most relevant. Where PBC is low, support should focus on practical barriers such as transportation, budgeting, reservations, and itinerary difficulty. Where weak Intention, an unfavorable Attitude, or low content appeal forms part of the relevant combination, simply providing additional factual information may be insufficient. The practical value of fsQCA therefore lies in identifying different combinations of barriers and enabling differentiated information and service strategies rather than searching for a single intervention applicable to all potential travelers.

Finally, the high-intensity and highly compressed nature of SFT means that related promotion should also incorporate traveler safety and well-being. Platforms, content creators, and destinations should avoid using heavily compressed editing or challenge-oriented narratives to imply that exceptionally intensive itineraries are appropriate for all young travelers. Content involving long walking distances, consecutive late-night activities, very early departures, or densely scheduled visits to multiple attractions can be accompanied by realistic duration estimates, rest recommendations, last-transport information, and pre-trip physical-readiness guidance. This recommendation concerns travelers’ assessment of their own physical capacity and anticipated demands before participation, rather than fatigue generated after participation has already begun. Embedding credibility, feasibility, and safety information into SFT promotion and product design may allow tourism organizations to respond to young travelers’ demand for efficient and intensive travel experiences while reducing risks arising from unrealistic information or itinerary expectations.

### 5.5 Limitations and future research

This study has several limitations that also provide directions for future research. First, the study employed a cross-sectional design in which Intention and self-reported behavior were measured within the same survey, although their temporal referents were not fully aligned. Intention primarily reflected respondents’ current plans, willingness, and readiness for future participation in SFT, whereas the BEH items primarily captured prior or habitual SFT-related travel patterns. Accordingly, H5 represents a cross-sectional statistical association between current Intention and BEH rather than evidence that Intention temporally preceded or caused subsequent behavior. In addition, outcome measurement relied on participants’ reports rather than objective records such as GPS traces, transportation or accommodation bookings, electronic ticketing data, or platform logs, and may therefore be affected by recall error, subjective interpretation, and social-desirability bias. Future research could adopt longitudinal designs in which Intention is measured when travel plans are formed and subsequent behavior is assessed several weeks or months later, while also incorporating objective behavioral records where possible [50].

Second, the explanatory scope of the current model for self-reported behavior remains limited. Its R^2^ was 0.263, indicating that 26.3% of its variance was explained while a substantial proportion remained outside the present specification. Although Intention and the Intention × SVPUI interaction were significantly associated with BEH, the findings should not be interpreted as providing a complete explanation of an intention–behavior gap. Future studies could incorporate additional real-world factors such as actual time availability, realized travel costs, transportation accessibility, coordination with travel companions, unexpected scheduling changes, and pre-existing physical readiness. Physical factors should be conceptualized as health or stamina constraints and anticipated physical demands that exist before participation, rather than fatigue generated after the trip has already begun.

Third, subjective norm was not measured, which is a limitation of model specification. In the original TPB, attitude, subjective norm, and PBC are distinct antecedents of intention [18]. The present focus on individual evaluations, feasibility, and digital-media perceptions does not demonstrate that friends’ or family members’ approval and expectations are unimportant. PCA, PCT, and SVPUI cannot substitute for subjective norm. Its independent association with intention could not be assessed, and we cannot determine whether including it would alter the reported path estimates, explanatory power, or configurational solutions. The results therefore apply to the selected constructs and should not be interpreted as a test of the complete TPB model. Future research should directly measure subjective norm, distinguish injunctive from descriptive norms where appropriate, and compare model specifications to evaluate the contribution of normative influence to SFT intention and behavior.

Finally, the external validity of the findings is limited by the population and geographic scope of the sample. The study focused exclusively on Chinese Generation Z university students, and the results should therefore not be generalized directly to the entire Chinese Generation Z population. Although university students are highly relevant to SFT, employed members of Generation Z may differ in income, leave arrangements, family responsibilities, and access to travel resources. Moreover, although the study used a multi-site sample from universities in Hunan and Guangdong Provinces to broaden geographic and institutional coverage, these two provinces cannot represent the full diversity of young travelers across China. Future research could compare university students with early-career professionals and extend sampling across eastern, central, and western China and different city tiers to assess whether the structural relationships and configurational patterns identified here remain stable across populations and regions.

## 6. Conclusion

This study examined survey data from 526 Chinese Generation Z university students and combined PLS-SEM with fsQCA within a modified Theory of Planned Behavior framework to investigate the relationships among short-video content perceptions, behavioral evaluation, perceived behavioral control, platform usage intensity, Intention, and self-reported SFT-related behavior. The PLS-SEM results showed that PCA, PCT, Attitude, and PBC were all positively associated with Intention, which was in turn positively associated with BEH. SVPUI also significantly moderated this association, which became stronger at higher levels of platform usage intensity. The fsQCA results further showed that no single condition was necessary for either high or low BEH. Two sufficient configurations were associated with high BEH and shared a relatively stable core consisting of Intention, PCT, PBC, and SVPUI. In contrast, low BEH was associated with a larger and more heterogeneous set of configurations, demonstrating clear configurational asymmetry rather than a simple reversal of the solutions for high BEH.

Overall, the findings suggest that understanding self-reported SFT-related behavior in short-video-mediated environments requires attention to both behavioral-level evaluations and feasibility and digital-content-related perceptions and platform engagement. PLS-SEM identifies the average net associations among these factors, whereas fsQCA shows that similar behavioral outcomes may be associated with different combinations of conditions. The two approaches therefore provide complementary rather than redundant perspectives on digitally mediated tourism behavior. The study consequently offers a more contextualized account of how short-video content evaluations, travel intention, and behavioral patterns are related among Chinese Generation Z university students. However, these conclusions apply primarily to the population examined in the present study, and their applicability to other Generation Z groups, regions, and cultural contexts remains to be established through future research.

## Supporting information

S1 Appendix Measurement items and sources. (DOCX)
