# Supplementary material for: Short-video content perceptions, intention, and self-reported behavior in special forces-style tourism: Evidence from Chinese generation Z university students
Source: PLoS One. 2026 Nov 3;21(11):e0361990. doi: 10.1371/journal.pone.0361990 (PMC13730330; doi:10.1371/journal.pone.0361990)
Supplement: Supplementary file 1 [file pone.0361990.s001.docx]

**S1 Appendix. Measurement items and sources.**

Measurement items and their sources for the seven constructs examined in the study. All items were assessed using a five-point Likert scale.

| **Construct** | **Code** | **Measurement item** | **Sources** |
| --- | --- | --- | --- |
| Attitude | ATT1 | I believe that participating in Special Forces-style tourism has a positive effect on my life. | [1, 2, 3] |
|  | ATT2 | I believe that participating in Special Forces-style tourism would be a pleasant experience. |  |
|  | ATT3 | I think Special Forces-style tourism is an exciting and rewarding experience. |  |
|  | ATT4 | In my opinion, Special Forces-style tourism is a novel, interesting and worthwhile form of travel to experience. |  |
| Intention | INT1 | If I have the opportunity, I would like to participate in Special Forces-style tourism. | [4, 5, 6, 7, 8] |
|  | INT2 | I plan to participate in Special Forces-style tourism within the next year. |  |
|  | INT3 | I intend to coordinate with friends or seek out information in preparation for Special Forces-style tourism. |  |
|  | INT4 | I am willing to prioritize Special Forces-style tourism in my travel plans. |  |
| Self-reported behavior | BEH1 | In the past year, I have taken at least one fast-paced trip similar to 'Special Forces-style' tourism (with tight scheduling and high intensity). | [9, 10] |
|  | BEH2 | In my travels, I tend to plan intensive itineraries, aiming to experience more attractions in a short time. |  |
|  | BEH3 | In my travels, I have engaged in physically demanding activities such as night tours, long hikes, or full-day itineraries from early morning to late at night. |  |
|  | BEH4 | My past travel experiences have typically involved tight schedules and high-intensity itineraries, rather than leisurely or relaxing trips. |  |
| Perceived Content Appeal | PCA1 | The Special Forces-style tourism short videos I have watched are visually engaging and emotionally stimulating. | [11, 12, 13] |
|  | PCA2 | The content of travel short videos usually has strong appeal, deeply moving me after watching. |  |
|  | PCA3 | The Special Forces-style tourism short videos I have watched are compelling enough that I find it hard to stop watching. |  |
|  | PCA4 | The Special Forces-style tourism short videos I have watched provide useful and practical information that helps me plan or imagine my own trip. |  |
| Perceived Content Trustworthiness | PCT1 | I think the content about “Special Forces-style tourism” in short videos is real and trustworthy. | [14, 15, 16] |
|  | PCT2 | I believe that the itineraries, costs, and modes of “Special Forces-style tourism” shown on short-video platforms basically match reality. |  |
|  | PCT3 | The Special Forces-style tourism content shown in short videos appears accurate and consistent with what travelers actually experience. |  |
|  | PCT4 | I trust that the travel content shared by short-video bloggers is based on real experiences, not fabricated or promotional. |  |
| Perceived Behavioral Control | PBC1 | As long as I want to, I can easily go on a Special Forces-style tourism trip. | [1, 5, 6, 17] |
|  | PBC2 | Whether I can smoothly undertake a Special Forces-style tourism trip mainly depends on myself. |  |
|  | PBC3 | I am confident I can overcome obstacles and successfully complete my Special Forces-style tourism plan. |  |
|  | PBC4 | I have enough time, money, and physical strength to arrange a Special Forces-style tourism trip. |  |
| Short-Video Platform Usage Intensity | SVPUI1 | I frequently notice and pay attention to likes, comments, and peer sharing about Special Forces-style tourism on short-video platforms. | [18, 19] |
|  | SVPUI2 | I often watch travel-related content on short-video platforms, especially about short trips and challenge-type travel. |  |
|  | SVPUI3 | I frequently search for a variety of travel-related short videos on short-video platforms to get inspiration for my trips. |  |
|  | SVPUI4 | I find it hard to imagine going without short-video platforms for a long time to get travel inspiration or for entertainment. |  |

# References

1. Ajzen I. The theory of planned behavior. Organ Behav Hum Decis Process. 1991;50(2):179–211. doi: 10.1016/0749-5978(91)90020-T.

2. Han H, Hsu LTJ, Sheu C. Application of the theory of planned behavior to green hotel choice: testing the effect of environmental friendly activities. Tour Manag. 2010;31(3):325–334. doi: 10.1016/j.tourman.2009.03.013.

3. Lam T, Hsu CHC. Predicting behavioral intention of choosing a travel destination. Tour Manag. 2006;27(4):589–599. doi: 10.1016/j.tourman.2005.02.003.

4. Hsu CHC, Huang S. An extension of the theory of planned behavior model for tourists. J Hosp Tour Res. 2012;36(3):390–417. doi: 10.1177/1096348010390817.

5. Han H, Meng B, Kim W. Emerging bicycle tourism and the theory of planned behavior. J Sustain Tour. 2017;25(2):292–309. doi: 10.1080/09669582.2016.1202955.

6. Soliman M. Extending the theory of planned behavior to predict tourism destination revisit intention. Int J Hosp Tour Adm. 2021;22(5):524–549. doi: 10.1080/15256480.2019.1692755.

7. Bui NA, Kiatkawsin K. Examining Vietnamese hard-adventure tourists’ visit intention using an extended model of goal-directed behavior. Sustainability. 2020;12(5):1747. doi: 10.3390/su12051747.

8. Yuzhanin S, Fisher D. The efficacy of the theory of planned behavior for predicting intentions to choose a travel destination: A review. Tour Rev. 2016;71(2):135–147. doi: 10.1108/TR-11-2015-0055.

9. Karl M, Bauer A, Ritchie WB, Passauer M. The impact of travel constraints on travel decision-making: A comparative approach of travel frequencies and intended travel participation. J Destin Mark Manag. 2020;18:100471. doi: 10.1016/j.jdmm.2020.100471.

10. Bui TTB. Applying the extended theory of planned behavior to understand domestic tourists' behaviors in post COVID-19 era. Cogent Soc Sci. 2023;9(1):2166450. doi: 10.1080/23311886.2023.2166450.

11. Tang YT, Ruan WQ, Zhang SN, Li X, Zhou Y. What makes you palpitate with excitement? The differentiation effect and mechanism of message appeal in tourism short-videos. J Travel Res. 2024. doi: 10.1177/00472875231225396.

12. Guo X, Liu B, He J, Li S. The impact mechanism of tourism short-video content marketing on users’ travel behavioral intention. Humanit Soc Sci Commun. 2025;12(1):494. doi: 10.1057/s41599-025-04801-3.

13. Liu J, Wang Y, Chang L. How do short videos influence users’ tourism intention? A study of key factors. Front Psychol. 2023;13:1036570. doi: 10.3389/fpsyg.2022.1036570.

14. Ayeh JK, Au N, Law R. “Do we believe in TripAdvisor?” Examining credibility perceptions and online travelers’ attitude toward using user-generated content. J Travel Res. 2013;52(4):437–452. doi: 10.1177/0047287512475217.

15. Kim M, Kim J. The influence of authenticity of online reviews on trust formation among travelers. J Travel Res. 2020;59(5):763–776. doi: 10.1177/0047287519868307.

16. Fu SX, Song JL, Deng SL, Zhu MD. The influence of semantic manipulation of multimodal content in fake short-videos on user trust. Library Journal. 2025;44(2):108–115. doi: 10.13663/j.cnki.lj.2025.02.012.

17. Erul E, Woosnam KM, McIntosh WA. Considering emotional solidarity and the theory of planned behavior in explaining behavioral intentions to support tourism development. J Sustain Tour. 2020;28(8):1158–1173. doi: 10.1080/09669582.2020.1726935.

18. Choi Y, Wen H, Chen M, Yang F. Sustainable determinants influencing habit formation among mobile short-video platform users. Sustainability. 2021;13(6):3216. doi: 10.3390/su13063216.

19. Ye JH, Zheng J, Nong W, Yang X. Potential effect of short-video usage intensity on short-video addiction, perceived mood enhancement (‘TikTok Brain’), and attention control among Chinese adolescents. Int J Ment Health Promot. 2025;27(3):271–286. doi: 10.32604/ijmhp.2025.059929.
